# Mesenchymal Stem Cell-Derived Antimicrobial Peptides as Potential Anti-Neoplastic Agents: New Insight into Anticancer Mechanisms of Stem Cells and Exosomes

**DOI:** 10.3389/fcell.2022.900418

**Published:** 2022-07-06

**Authors:** Kasra Moeinabadi-Bidgoli, Malihe Rezaee, Hamidreza Rismanchi, Mohaddese Malek Mohammadi, Amirhesam Babajani

**Affiliations:** ^1^ Department of Pharmacology, School of Medicine, Shahid Beheshti University of Medical Sciences, Tehran, Iran; ^2^ Basic and Molecular Epidemiology of Gastroenterology Disorders Research Center, Shahid Beheshti University of Medical Sciences, Tehran, Iran; ^3^ School of Medicine, Shahid Beheshti University of Medical Sciences, Tehran, Iran; ^4^ Tehran Heart Center, Cardiovascular Diseases Research Institute, Tehran University of Medical Sciences, Tehran, Iran

**Keywords:** mesenchymal stem cell, antimicrobial peptides, exosomes, apoptosis, angiogenesis, cell cycle, multidrug resistance

## Abstract

Mesenchymal stem cells (MSCs), as adult multipotent cells, possess considerable regenerative and anti-neoplastic effects, from inducing apoptosis in the cancer cells to reducing multidrug resistance that bring them up as an appropriate alternative for cancer treatment. These cells can alter the behavior of cancer cells, the condition of the tumor microenvironment, and the activity of immune cells that result in tumor regression. It has been observed that during inflammatory conditions, a well-known feature of the tumor microenvironment, the MSCs produce and release some molecules called “antimicrobial peptides (AMPs)” with demonstrated anti-neoplastic effects. These peptides have remarkable targeted anticancer effects by attaching to the negatively charged membrane of neoplastic cells, disrupting the membrane, and interfering with intracellular pathways. Therefore, AMPs could be considered as a part of the wide-ranging anti-neoplastic effects of MSCs. This review focuses on the possible anti-neoplastic effects of MSCs-derived AMPs and their mechanisms. It also discusses preconditioning approaches and using exosomes to enhance AMP production and delivery from MSCs to cancer cells. Besides, the clinical administration of MSCs-derived AMPs, along with their challenges in clinical practice, were debated.

## Introduction

Cancer as an invasive heterogeneous disease is still a significant health problem and a barrier to increasing life expectancy worldwide. According to the World Health Organization (WHO), neoplasms have been the first or second leading cause of mortality in many countries during the last 7 years (Sung et al., 2021). Although the establishment of therapeutic approaches such as chemotherapy, radiotherapy, surgical tumor resection, and neoadjuvant therapy has reduced cancer mortality, the impressive side effects, expensive costs, and drug/equipment unavailability have undermined the cost-effectiveness of these treatments (Franzoi et al., 2021; Nolsøe et al., 2021). One of the most critical challenges in cancer treatment is the non-targeted therapeutic manner which causes collateral toxicity and intolerable side effects. Thus, many approaches have been developed to prioritize targeting tumors with less toxic effects on non-cancerous body tissues (Ye et al., 2021).

Using mesenchymal stem cells (MSCs) is one of the promising approaches for targeted cancer therapy. MSCs are self-renewing multipotent stem cells with great potential to differentiate into several cells such as adipocyte, chondrocyte, osteocyte, and myocyte. Thus, they are considered promising therapeutic approaches for regenerative medicine and tissue engineering (Alcayaga-Miranda et al., 2015a). Tissue Stem Cell Committee of the International Society for Cellular Therapy (ISCT, www.celltherapysociety.org) has suggested standard criteria for distinguishing human MSCs, including 1) the ability to attach to the plastic surface; 2) the presence of some specific surface markers (positive expression of CD105, CD73, and CD90, and lack expression of CD45, CD34, CD14 or CD11b, CD79a or CD19, and HLA-DR); and 3) multipotent differentiation potential to different cell types including osteoblast, adipocytes, and chondroblasts using standard *in vitro* tissue culture-differentiating conditions (Dominici et al., 2006). MSCs show minimal transcription and translation of immunogenic markers, major histocompatibility complex (MHC)-I and MHC-II, suggesting immune evasion and lower rejection chance in MSC transplantation (Huaman et al., 2019). َAditionally, MSCs migrate and home into the tumor microenvironment (TME) mainly through three mechanisms: detecting specific cytokines and chemokines in TME, expressing chemokine receptors and adhesion molecules, and exclusive metabolic status of TME (Dwyer et al., 2007; Spaeth et al., 2008). They also have intrinsic anti-neoplastic hallmarks on tumors, such as inducing apoptosis, inhibiting proliferation, reducing angiogenesis, and preventing metastasis (Babajani et al., 2020). Discovering different anti-tumor mechanisms of MSCs will shed light on the possible application of MSCs in various neoplastic conditions and allow scientists to enhance and modify these anti-neoplastic traits. Thus, several studies attempt to declare the underlying mechanisms of anti-tumor activity of MSCs (Qiao et al., 2008; Bhoopathi et al., 2011; Mangraviti et al., 2016; Lu et al., 2019).

The growing evidence elucidated that MSCs’ secretome contains broad-range molecules mainly incorporated in small extracellular vesicles (EVs). Exosomes are the leading group of EVs that play an essential role in intercellular communication, biologic processes, immunomodulation, apoptosis, and angiogenesis by carrying and transferring several molecules such as messenger RNAs (mRNAs), microRNAs, DNAs, proteins, and lipids (Nawaz et al., 2016). In this regard, recent studies have reported that MSCs release a class of small peptides called “antimicrobial peptides (AMPs)” (Harman et al., 2017; Yagi et al., 2020). These peptides play critical roles as the first line of immune defense against various organisms, including bacteria, fungi, and viruses (Brogden, 2005; Zhang and Gallo, 2016). Although most preclinical and clinical studies have focused on the antimicrobial properties of AMPs, many recent pieces of research have proposed that AMPs also have targeted anti-neoplastic activity (Elrayess et al., 2020; Su and Chen, 2020; Swithenbank et al., 2020). AMPs specifically target cancer cells and induce various anticancer effects by disrupting the plasma membrane, interfering with intracellular molecular pathways, affecting the mitochondrial membrane, altering TME, and affecting immune responses. Consequently, AMPs promote apoptosis/necrosis, attenuate proliferation, angiogenic, metastasis, and multidrug resistance (MDR) in tumors (Chavakis et al., 2004; Wang et al., 2013; Kuroda et al., 2015; Jiang and Lönnerdal, 2017; Norouzi et al., 2018; Lv et al., 2019).

Considering the fact that efficient treatment responses depend on the interaction of the therapeutic agents with cancer cells and TME, focusing on the ability of MSCs to produce and release AMPs and the anticancer role of AMPs in TME could shed light on new anticancer mechanisms of MSCs (Wheeler et al., 2021). This review summarizes the possible application of MSCs-derived AMPs regarding their anticancer function. It also discusses different mechanisms of anti-neoplastic effects of these AMPs. We also underlined the presence of AMPs in the cargo of MSC-derived exosomes, the proposed role of preconditioning in increasing therapeutic effects of MSC-derived AMPs, and also translational challenges of AMPs into clinical practice.

## Characteristics of Antimicrobial Peptides

Antimicrobial peptides are a class of small host defense peptides (10–150 amino acids) found in various organisms, from prokaryotes to humans (Zhang and Gallo, 2016). According to the AMP database, 3,324 AMPs have been recognized up to March 2022, among which 261 AMPs are listed as anticancer peptides (www.aps.unmc.edu). AMPs exhibit extraordinary physicochemical diversity in properties that construct their exclusive activities. These features mainly depend on amino acid sequences, length of the peptides, electrostatic charge of the molecules, lipid composition, hydrophobicity, amphipathicity, and spatial structural folding, including secondary structure, dynamics, and orientation (Jenssen et al., 2006; Hoskin and Ramamoorthy, 2008; Li et al., 2021). The majority of AMPs are amphipathic peptides that show a positive net charge with a significant proportion of hydrophobic residues at neutral pH. The balance between charge distribution and hydrophobicity of AMPs plays an essential role in their function (Melo et al., 2011; Chu et al., 2015; Deslouches and Di, 2017).

AMPs could be classified into different categories based on the various properties such as electrostatic charge, structure, amino acid components, mode of action, and origin (Lei et al., 2019). From the secondary structural point of view, AMPs are classified into four categories: α-helix, β-sheet, extended or random coil, and cyclic or loop peptide (Rajchakit and Sarojini, 2017; Xie et al., 2020). The α-helix AMPs are the most extensively studied class with random conformations in aqueous solutions while possessing a helical conformation during interaction with cell membranes (Tornesello et al., 2020). Typical examples of the α-helix peptides are human cathelicidin LL-37, histatins, dermcidin, and granulysin (Wang, 2014). The β-sheet AMPs are characterized by at least two β-strands containing one or more disulfide cysteine-cysteine bonds that stabilize the structure and facilitate cell membrane penetration (Wu et al., 2018; Seyfi et al., 2020). Human α-defensins and hepcidins are examples of β-sheet AMPs (Wang, 2014). Extended AMPs, non-αβ peptides, do not fold into regular secondary structures. They often comprise a high percentage of specific amino acids, ineffective against cell membranes (Nguyen et al., 2011). The cyclic peptides are the smallest group of AMPs that form closed-loop structures composed of head-to-tail cyclization or disulfide bonds (Xie et al., 2020).

AMPs are critical components of the innate immune response that defend different organisms by inducing a wide range of inhibitory effects during the initial stages of infection (Ganz, 2003). They display immune responses against various microorganisms, such as viruses, Gram-positive and Gram-negative bacteria, and fungi. Although the molecular mechanisms by which they act are not yet fully elucidated, their direct effect on the bacterial cell membrane is the most prevalent known activity of AMPs (Huerta-Cantillo and Navarro-García, 2016; Lee et al., 2019). In most scenarios, it is notable that the initial interaction with the plasma membrane through electrostatic charges is required (Huerta-Cantillo and Navarro-García, 2016). In order to describe the basis of electrostatic interaction of AMPs with the cell membrane, it has been shown that unlike the outer leaflet of the normal eukaryote cell membrane that mainly consists of zero net charged lipids, the outer side of the bacterial membrane contains a higher proportion of lipids with a negative charge such as lipopolysaccharide (LPS) in Gram-negative bacteria and teichoic and teichuronic acids in Gram-positive bacteria. Therefore, the cationic surface charges of AMPs are responsible for the electrostatic interactions and binding between AMPs and negatively charged lipids on the target cell membranes (Li et al., 2017).

After effective AMP-membrane interaction, AMPs’ mechanisms of action could be divided into two categories: membrane disruption and non-membrane disruption. In the membrane disruption mechanism, AMP-membrane interaction disrupts the bacterial membrane, causing an alteration in membrane permeability, formation of pores, lysis of the membrane, and cytoplasmic metabolite leakage (Lee et al., 2016; Browne et al., 2020; Jiang et al., 2021). Although most AMPs were described as potent peptides able to penetrate the membrane, their mechanisms of action are not limited to the cell membrane of the cells. In non-membrane disruption mechanisms, some AMPs interact with intracellular targets, such as inhibiting nucleic-acid and/or protein synthesis and/or enzymatic activity (Brogden, 2005; Yount and Yeaman, 2005; Hale and Hancock, 2007; Li et al., 2021). As a piece of evidence, AMPs interact with genomic DNA that directly affects the expression and synthesis of cellular metabolic substances (Bechinger and Gorr, 2017). It has been shown that PR-39 and defensin activate cytotoxic pathways *via* arresting DNA synthesis and breaking single-strand DNA, respectively (Kamysz et al., 2003). Similarly, some synthesized peptides, including KW4 and L5W, have been found to invade the pathogens by interacting with DNA. DP7, another synthetic AMP, causes alterations in nucleic acid metabolism and amino acid biosynthesis (Li et al., 2021). Besides, AMPs inhibit protein synthesis through interaction with bacterial 70s and 50s ribosomes. In this way, class I proline-rich AMPs block the delivery of AA-tRNA to the ribosomal A-site, whereas class II proline-rich AMPs act by trapping factors on the 70S ribosome following hydrolysis of the nascent polypeptide. (Li et al., 2021). Besides the direct defensive effects on infectious microorganisms, AMPs have more diverse roles in developed animals, including regulation of the innate and adaptive immune pathways, stimulation of chemokine and cytokines production, chemotactic activity, and modulation of Toll-like receptors (TLR)-dependent inflammatory responses (Lai and Gallo, 2009; Zhang and Gallo, 2016). Some defensins and LL-37 have potent immunomodulatory and immunoadjuvant activities, including chemotactic effects, complement activation, mast cell degranulation, and enhancing cytokine and chemokine production (Oppenheim et al., 2003; Meyer and Harder, 2007).

Besides the critical role of AMPs in defending against infections, various studies indicated that these molecules could have therapeutic effects on inflammatory disorders, autoimmunity, wounds, and malignancies (Luong et al., 2020). In this regard, a group of AMPs has been discovered that displayed anti-neoplastic activity, so-called “anticancer peptides (ACPs)” (Mader and Hoskin, 2006; Hoskin and Ramamoorthy, 2008; Jin and Weinberg, 2019). ACPs exhibit selective cytotoxic activity against a wide range of human cancer cells, including neoplastic cells that have acquired a MDR phenotype. The anti-neoplastic activities of α-helical ACPs, including some cathelicidins (BMAP-27, BMAP-28, and LL-37/hCAP-18), cecropin A and B, and melittin as well as β-Sheet ACPs including, defensins, lactoferricin, and tachyplesin were revealed in previous studies (Mader and Hoskin, 2006; Hoskin and Ramamoorthy, 2008; Jin and Weinberg, 2019). Besides, production and secretion of AMPs have been reported in a group of adult multipotent stem cells, MSCs (Sutton et al., 2016; Alcayaga-Miranda et al., 2017; Harman et al., 2017; Esfandiyari et al., 2019; Russell et al., 2020). Considering intrinsic anti-neoplastic activity of MSCs, including inducing apoptosis, inhibiting cancer cell proliferation, preventing angiogenesis, and inhibiting metastasis, discussing the possible role of ACPs in anti-neoplastic features of MSCs will shed light on newer anticancer approaches (Babajani et al., 2020).

## Mesenchymal Stem Cells as a Source of Antimicrobial Peptides

As self-renewing adult multipotent stem cells, MSCs could be isolated from various adult tissues, including bone, adipose tissue, synovium, dermis, periodontal ligament, dental pulp, amniotic membrane, and the umbilical cord (Nancarrow-Lei et al., 2017). Besides the regenerative ability, the therapeutic potential of MSCs in various pathological conditions such as infections, autoimmune diseases, and cancer has been established (Rad et al., 2019; Hmadcha et al., 2020; Yagi et al., 2020). One of the most promising therapeutic aspects of MSCs is anti-tumor activities. Antiproliferative effects, angiogenesis suppression, regulating metabolisms, and inducing apoptosis are the leading capabilities of the MSCs to combat neoplasms (Rhee et al., 2015). Additionally, MSCs efficiently migrate and home into the primary tumor and secondary metastasis sites due to the secretion of various chemoattractant molecules in the TME, including interferon (IFN)-γ, tumor necrosis factor-α (TNF-α), interleukin (IL)-6, IL-8, transforming growth factor (TGF)-β, hepatocyte growth factor (HGF), platelet-derived growth factor (PDGF), vascular endothelial growth factor (VEGF), and CXCL12 whose receptors exist on MSCs membrane (Dwyer et al., 2007; Seo et al., 2011; D’souza et al., 2013).

MSCs induce their therapeutic effects by producing and releasing various bioactive molecules such as TGF-β, IL-10, TNFα-stimulated gene-6 (TSG6), indoleamine-2,3-dioxygenase (IDO), and prostaglandin E2 (PGE-2) (Waterman et al., 2010). To the best of our knowledge, MSCs also produce multiple AMPs, including the cathelicidin peptide LL-37, hepcidin, human β-defensin-2 (hBD-2), and lipocalin-2 (Lcn2), which are described in Table 1. MSCs secrete AMPs in specific conditions based on the presence of determined immune mediators and/or antigens. For instance, exposure to bacteria induces the production of hBD-2, and hepcidin, while inflammatory conditions increase levels of Lcn2 in MSCs. Notably, both bacterial exposure and inflammatory condition increase the LL-37 level (Alcayaga-Miranda et al., 2017). Major innate immune pathways like TLRs, NOD-like receptors, and cytokines activate the MSCs to secrete bactericidal factors such as AMPs (Chow et al., 2020). Besides, inflammatory pathological conditions like systemic lupus erythematosus (SLE) and pulmonary diseases influence the expression of LL-37 in MSCs (Alcayaga-Miranda et al., 2017). Overall, inflammation seems to be a potent inducer of AMPs secretion from MSCs.

**TABLE 1:** Characteristics of MSC-derived AMPs with their antimicrobial effects.

AMP	Structure	MSCs source	Affected bacteria	References
Cathelicidin LL-37	α-helix	Human bone marrow	*Escherichia coli*, *Pseudomonas aeruginosa*, *Staphylococcus aureus*	Krasnodembskaya et al. (2010)
		Human bone marrow	*Pseudomonas aeruginosa*, *Staphylococcus aureus*	Sutton et al. (2016)
		Human adipose tissue		
		Human adipose tissue	*Staphylococcus aureus*	Yagi et al. (2020)
		Equine peripheral blood	*Escherichia coli*, *Staphylococcus aureus*	Harman et al. (2017)
		Murine adipose tissue	*Staphylococcus aureus*	Johnson et al. (2017)
		Murine bone marrow	*Mycobacterium* smegmatis, *Mycobacterium* bovis	Naik et al. (2017)
β-defensin	β-sheet	Human umbilical cord blood	*Escherichia coli*	Sung et al. (2016)
Hepcidin	β-sheet	Human menstrual blood	Antimicrobial activity in sepsis	Alcayaga-Miranda et al. (2015b)
Lipocalin-2	non-αβ	Murine bone marrow	*Escherichia coli*	Gupta et al. (2012)

Inflammation plays a central role in different stages of tumorigenesis and the malignant progression of developing cancer. In the first stages of tumorigenesis, inflammation triggers several intracellular pathways that increase the proliferation of existing cells, such as epithelial cells. Besides, oncogene-derived stress triggers the initiation of inflammation in TME. In that case, the inflammatory responses will last in a feed-forward loop of inflammatory signalings, promoting cancer progression in all stages. Besides, various anti-neoplastic therapies such as chemotherapy and radiotherapy develop inflammatory responses in TME that aids tumor progression (Greten and Grivennikov, 2019; Hou et al., 2021). It seems that persistent inflammation of TME can be a potent inducer for the secretion of AMPs from MSCs. Considering the anti-neoplastic effects of MSCs and the presence of several inflammatory mediators in TME, it could be proposed that secretion of AMPs in TME is regarded as one of the anticancer mechanisms of MSCs.

## Proposed Anticancer Effects of Mesenchymal Stem Cells-Derived Antimicrobial Peptides

Mesenchymal stem cells are supposed as producing factories of AMPs that attack malignant cells in a targeted manner. As mentioned above, the first step of AMPs action depends on the interaction between these peptides and the target malignant cells’ membrane. Biological membranes consist of two phospholipid layers with amphipathic properties, containing both hydrophobic and hydrophilic molecules. An intact healthy membrane usually has zwitterionic amphiphile distribution in which the outer surface remains neutral (Devaux, 1991; Li, 2015). On the other hand, it has been observed that altered microenvironmental conditions within the tumor, including hypoxia and increased reactive oxygen species (ROS), induced dysregulation of phospholipid transporters which changed the typical phospholipids pattern of the plasma membrane (Ran et al., 2002). In this regard, anionic phospholipids, including phosphatidylethanolamine (PE) and phosphatidylserine (PS), migrate from the inner side of the cancer cell membrane to the outer side, resulting in a negative charge of the outer membrane. This phenomenon increases the interaction of cationic AMP and anionic cancer cell membranes (Ran and Thorpe, 2002; Balasubramanian and Schroit, 2003). After peptide-membrane interaction, AMPs pass through the cell’s membrane (Park et al., 2000). Following the entrance of AMPs to the neoplastic cell, they induce various anticancer effects via promoting apoptosis, inhibiting proliferation, preventing angiogenesis, modulating immune responses, and reducing MDR.

### Promoting Apoptosis and Cell Death

AMPs induce cell death in various cancer cell types, such as urinary bladder cancer (Suttmann et al., 2008), breast cancer (Guzmán-Rodríguez et al., 2016), colorectal cancer (Norouzi et al., 2018), glioblastoma (Chen et al., 2020), non-small-cell lung carcinoma (NSCLC) (Liu et al., 2017), and multiple myeloma (Hilchie et al., 2013a). AMPs with the most anticancer potency are α-helical or β-sheet. As previously mentioned, MSCs secrete various α-helical or β-sheet AMPs, including LL-37, hepcidin, and hBD-2 (Liu et al., 2015; Alcayaga-Miranda et al., 2017). AMPs which MSCs can release, induce cell death through two mechanisms: 1) membranolytic, in which the disintegration of the cancer cell membranes and/or mitochondrial membranes results in cell death. 2) non-membranolytic interactions of AMPs with intracellular pathways triggering cell death pathways (Liu et al., 2016; Leite et al., 2018).

As the first mechanism, selective membrane disruption could achieve more anticancer impact with lower side effects. AMPs disrupt cell membranes through several action models, including barrel stave, toroidal, carpet, detergent, sinking raft, molecular electroporation, peptide-induced lipid segregation, and leaky-slit model (Liu et al., 2015; Tornesello et al., 2020). However, barrel stave and toroidal methods are more remarkable in MSCs-derived AMPs. In the barrel stave model, hydrophobic amino acids of AMPs invade the lipid bilayer’s hydrophobic core, stopping contact of the hydrophilic parts of the AMPs to the hydrophobic regions of the inner membrane. At this point, the hydrophobic parts of the peptide are exposed to the acylic chains of the membrane, leading to trans-membrane pores formation, leakage of cellular cytoplasm, and cell death. The pore formation process in the toroidal model is similar to the barrel stave model, except both peptides and lipids have critical roles in which AMPs dispose perpendicularly to the bilayer membrane and irreversibly destabilize membranes while preserving integrity (Liu et al., 2015). As a result, the lipid bilayer pores are formed within the cell membrane, and the entry of peptides into the inner membrane leaflet occurs via these toroidal pores (Tornesello et al., 2020). Indeed, many β-sheet AMPs, such as human α-defensins and hepcidins, disrupt cell membranes through toroidal pores formation (Nguyen et al., 2011). Besides, it has been shown that the LL-37 disintegrates cancer cell membranes via a toroidal pore mechanism, too (Xhindoli et al., 2016).

On the other hand, some MSCs-derived AMPs pass the membrane and induce cell death by accessing the intracellular compartments such as nucleic acids and organelles. Considering the bacterial origin of mitochondria and anionic phospholipids on the eukaryotic mitochondrial membranes, AMPs disrupt the mitochondria, resulting in mitochondrial membrane degradation and mitochondrial swelling, phosphatidylserine translocation to the cell surface, and apoptotic markers stimulation. Dysregulation of the mitochondrial membrane potential (ΔΨm) is a central intracellular trigger point for inducing apoptotic cell death (Daum, 1985; Zamzami et al., 1995; Deslouches and Di, 2017). For instance, LL-37 causes the release of cytochrome c, which activates the apoptotic protease activating factor 1 (APAF1). This proapoptotic factor cleaves and activates the pro-enzyme of caspase-9 that enhances the translocation of caspase-9 from mitochondria into the cytosol and consequently apoptosis (Li et al., 1997; Mader et al., 2009). On the other hand, as a mitochondria-related pathway (but independent of caspase), it has been shown that LL-37 meaningfully induced Bax relocation to mitochondria, where it can lead to ΔΨm dissipation and translocation of apoptosis-inducing factor (AIF) from intermembrane space of the mitochondria to the nucleus in Jurkat T leukemia cells. Increased levels of these proapoptotic factors in the nucleus caused chromatin condensation, DNA degradation, and programmed cell death as early as 4 h after LL-37 administration (Mader et al., 2009; Sevrioukova, 2011). Besides, apoptotic stimuli enhance lysosomal membrane permeability and resultant release of proapoptotic lysosomal cathepsins, which finally initiate cytosol death signaling pathways (Oberle et al., 2010). It seems that LL-37 can also cause cell death through calcium- and calpains-mediated lysosomal permeabilization (Mader et al., 2009). Along with these studies, it has been observed that the active domain of LL-37 induced mitochondrial depolarization, which triggered apoptosis in a caspase-independent manner in human oral squamous cell carcinoma (OSCC) (Okumura et al., 2004). Interestingly, it has been shown that MSCs induced mitochondrial dysfunction by increasing the BAX/Bcl-2 and BAX/Bcl-xL ratio and dysregulating the mitochondrial membrane potential, resulting in initiation of the intrinsic apoptosis pathway (Bu et al., 2016).

The anticancer roles of other AMPs assumed to be released from MSCs have been evaluated. For instance, treatment of fibrosarcoma cells with hepcidin resulted in downregulation of c-Jun mRNA expression, as the most extensively studied protein of the activator protein-1 (AP-1) complex (Chen et al., 2009). It has been shown that c-Jun participated in tumor initiation and cancer progression by antagonizing tumor-suppressive effects of TP-53 (Eferl et al., 2003). In another study, hepcidin combined with epirubicin, an anthracycline chemotherapeutic drug, induced apoptosis via the reducing mitochondrial membrane potential and increasing reactive oxygen species (ROS) accumulation inside the human squamous cell carcinoma and human embryonal carcinoma cells (Lo et al., 2015). The presence of ROS can trigger cancer cell death through various mechanisms, including DNA damage which activates TP53, and increasing BAX pro-apoptotic factor, which activates mitochondrial-dependent apoptotic pathways (Juang et al., 2016).

As another non-membranolytic anticancer effect, AMPs interact with cancer cell metabolism, promoting cell death. As the well-known metabolic feature of neoplastic cells, glycolysis is responsible for most ATP generation rather than oxidative phosphorylation in cancer cells called the “Warburg effect” (Liberti and Locasale, 2016). It has been shown that FF/CAP18, an analog peptide of LL-37, suppresses ATP generation by reducing the level of fructose 6-phosphate to fructose 1,6-bisphosphate conversion, the rate-limiting step of glycolysis, and enhanced pentose phosphate pathway. This metabolic alteration can be attributed to the effects of TP53-inducible glycolysis, which blocks glycolysis at this stage (Kuroda et al., 2015). It seems that MSCs-derived AMPs could potentially enhance cell death by disrupting cancer cell membrane and different mitochondrial and non-mitochondrial pathways.

### Inhibiting Proliferation

Cancer cell uncontrolled proliferation is a well-established characteristic of tumors mediated by altered expression and/or activity of cell cycle-related proteins. Several intracellular molecules regulate the cell cycle, including the family of cyclin-dependent kinases (CDKs), their inhibitors, cyclins as regulatory subunits of CDK, the retinoblastoma (RB) family members, and the E2F transcription factors (Huber et al., 2021). In this regard, MSCs regulate the expression of cell cycle-related molecules, preventing cancer cell transition between different cell phases and resultant cancer cell cycle arrest. MSCs down-regulate cell cycle positive regulators such as CCND2, CDK2, CDK4, CDK6, CUL1, SKP2, RBL1, CCNE, CCNH, which control the G1 phase and G1/S transition. Besides, they are able to suppress the G2 phase and G2/M growth by regulating CCNH, CDK5R1, and DDX11 (Magatti et al., 2012; Bu et al., 2016).

AMPs, especially AMPs released from MSCs, induce cancer cell cycle arrest as a critical anti-neoplastic feature. It has been shown that LL-37 and FF/CAP18, its analog peptide, increased the levels of miR-663a in colorectal cancer cells. Notably, MicroRNAs degrade mRNA, resulting in mRNA translation suppression. LL-37-associated raised miR-663a attaches to the coding sequence of CXCR4 mRNA that suppresses CXCR4 translation and consequent lowering phosphorylated protein kinase B (Akt). This pathway ultimately activates p21, inducing cycle arrest at the G2/M phase and tumor cell growth suppression (Kuroda et al., 2017). In another study, LL-37 triggered the tumor-suppressing bone morphogenetic protein (BMP) signaling via increasing BMP4 expression and subsequent Smad1/5 phosphorylation as the downstream of the BMP signaling pathway. This signaling finally induced p21 activation and G1/S transition delay (Wu et al., 2010).

On the other hand, some studies have shown that AMPs inhibit cancer cell proliferation regardless of cancer cell cycle regulatory proteins. For instance, FF/CAP18, an analog of LL-37, significantly reduced the proliferation of colon cancer cells in a dose-dependent manner. Interestingly, the anti-proliferative effect of FF/CAP18 was independent of TP53, as a tumor suppressor protein that induces G2/M phases arrest (Kuroda et al., 2012; Chen, 2016). In this regard, the impact of AMPs on TME was evaluated to shed light on their anti-proliferative effects. Cheng et al. found that cathelicidin bind tubulin proteins of cancer-associated fibroblasts and disrupts cytoskeletal tubulin. Several studies have shown that cancer-associated fibroblasts promote tumor progression in various cancer types. They demonstrated that AMPs indirectly interfere with fibroblast-induced proliferation in colon cancer cells by destroying the fibroblast cytoskeleton *in vivo*. Interestingly, the mechanisms of these anti-proliferative effects are similar to microtubule stabilizers chemotherapeutic agents like taxol and paclitaxel (Orr et al., 2003; Cheng et al., 2015b; Sahai et al., 2020). Collectively, MSCs-derived AMPs can inhibit cancer cell proliferation by regulating cell cycle checkpoints and/or modulating TME.

### Inhibiting Angiogenesis

Angiogenesis is sprouting new vessels from pre-existing capillaries combined with a longitudinal extension of pre-existing vessels (Kanazawa et al., 2019). The progression of tumor cells is limited to 1–2 mm^3^ without angiogenesis potential, while this size expansion is indicated to be more than 2 mm^3^ in the presence of angiogenesis ability and appropriate blood circulation showing the particular importance of angiogenesis in the proliferation of cancer cells (Nishida et al., 2006). Angiogenesis and lymphangiogenesis are induced by chemical signals from tumor cells in the rapid growth phase during tumor progression (Oshi et al., 2021). Different proteins participate in the angiogenesis process, including VEGF, basic fibroblast growth factor (bFGF), angiogenin, TGF-α, TGF-β, TNF-α, platelet-derived endothelial growth factor (PDGF), granulocyte colony-stimulating factor (G-CSF), placental growth factor, IL-8, hHGF, and epidermal growth factor (EGF) (Folkman, 1995; Appelmann et al., 2010; Voron et al., 2014). These pro-angiogenic factors accelerate the transition from one stage to another during the angiogenesis process, including protease production, migration and proliferation of endothelial cells, vascular tube formation (canalization), anastomosis of newly formed vascular tubes, construction of a new basement membrane, and attachment of pericytes and smooth muscle cells (Rajabi and Mousa, 2017).

Mesenchymal stem cells have anti-angiogenic effects by inducing apoptosis in endothelial cells, inhibiting pro-angiogenic factors, and impeding migration in endothelial cells. Direct contact of endothelial cells and MSCs leads to the transfer of mitochondria of MSCs to endothelial cells, increasing ROS products in endothelial cells and consequently inducing apoptosis (Otsu et al., 2009). Besides, MSCs up-regulate the caspase-3 and persuade the FasL-associated pathway in endothelial cells in order to encourage apoptosis and prevent angiogenesis (Babajani et al., 2020). Moreover, MSC-derived exosomes inhibit the expression of VEGF in TME via their microRNA-16 content (Lee et al., 2013). As a point of interest, some pieces of evidence have shown that MSCs-derived AMPs also prevent angiogenesis in TME. It has been observed that defensins could inhibit the migration of endothelial cells. Furthermore, defensins impede the formation of capillary-like tubes *in vitro* by blocking either av- or β1-integrin (Kougias et al., 2005). Defensins also block VEGF-induced proliferation and VEGF- and bFGF-induced capillary formation ability of endothelial cells (Economopoulou et al., 2005). Hanaoka *et al.* have shown that infusion of defensin into Lewis lung carcinoma cells in mice significantly decreased the tumor size by suppressing angiogenesis in the animal model without damaging normal cells around the infusion site (Hanaoka et al., 2016). It seems that defensins could be considered an endogenous anti-angiogenic factor that modulates the balance between pro-angiogenic and anti-angiogenic agents in pathologic conditions (Economopoulou et al., 2005).

As another anti-angiogenic example of MSCs-derived AMPs, Fan et al. have invented a new drug delivery platform for colorectal cancer in which a biodegradable and injectable nanoparticle–hydrogel composite of docetaxel and LL37 was administered. This approach reduced microvessel density in a colorectal peritoneal carcinomatosis mouse model, which showed improved results compared to pure docetaxel alone (Fan et al., 2015). Besides, it has been observed that LL-37 induces vascular smooth muscle cell apoptosis *via* increasing the plasma membrane permeability (Ciornei et al., 2006). Altogether, AMPs could disturb angiogenesis and prevent tumor growth and invasion via inducing hypoxia and nutrition poverty in the tumor environment.

### Immunomodulation

Mostly, the immune system plays an essential role in controlling the growth of tumoral cells. Recognition of tumor antigens by the immune system evokes immune responses and release of various cytokines in order to prevent tumor progression. If the immune response were effective, it could eliminate the malignant cells by phagocytosis and/or activation of apoptotic pathways. While the immune reaction isn’t adequate, malignant cell clones emerge and grow (Dunn et al., 2004). Cytotoxic CD8^+^ T cells, as an adaptive immune system component, are the leading killers of pathogens and neoplastic cells by secreting cytokines, such as IFN-γ and TNF, or cytotoxic molecules such as granzymes and perforin. Cytotoxic CD8^+^ T cells cooperate with CD4^+^ T cells to maintain the CD8^+^ responsiveness and prevent CD8^+^ hyposensitivity (Philip and Schietinger, 2021; Raskov et al., 2021). As a part of innate immunity, macrophages play various roles ranging from anti-tumor activity in early progression stages to tumor-promoting roles in established cancer (Noy and Pollard, 2014). Classically there are two macrophage phenotypes, including M1 and M2. Infiltration of the M2 subtype in the tumor environment corresponds with the poor outcome due to pro-tumorigenic functions, while M1 subtype infiltration contributes to a desirable outcome due to antitumorigenic effects (Pollard, 2004; Bruni et al., 2020).

AMPs have very complex immunomodulatory influences via regulating the secretion of immune mediators, the activities of immune cells, the cell surface receptors, and several intracellular signal pathways (Zhang Q.-Y. et al., 2021). It has been observed that AMPs selectively modulate innate immune pathways and inflammatory responses (Gardy et al., 2009). The binding of AMPs to intracellular receptors activates transduction signals important in innate immunity, including p38, extracellular related kinases 1 and 2 (ERK1/2), JNK mitogen-activated protein kinases (MAPKs), nuclear factor-kB (NF-kB), PI3K, three Src family kinases, TRIF–interferon regulatory factor (IRF), and TREM (Hilchie et al., 2013b). As a part of innate immunity, M2 and M1 macrophages are protumorigenic and antitumorigenic, respectively. M1 macrophages suppress tumor growth through phagocytosis and cytokine secretion such as IFN-γ, IFN-β, and IFN-α. Several studies showed that LL-37 activates the M1 phenotype of macrophages (Mookherjee et al., 2009; Fabisiak et al., 2016). In this regard, the animal models of pancreatic cancer treated with LL-37 demonstrate a significantly decreased expression of the M2 macrophage, and an adequate concentration of LL-37 in TME caused pancreatic tumor growth inhibition (Ko and Zhang, 2019). In addition to LL-37, Lcn-2 suppresses the macrophage M2 phenotype and improves polarization into the M1 phenotype (Cheng et al., 2015a). Besides, one of the immunomodulatory roles of LL-37 that induces a desirable immune system response is triggering ROS production in neutrophils, which is a defense mechanism to eliminate and prevent the progression of cancer cells (Esfandiyari et al., 2019; Wu et al., 2019).

It has been demonstrated that LL-37 induces the differentiation of dendritic cells and enhances the production of INF-γ from T cells (Davidson et al., 2004). T cell-derived IFN-γ activates the anti-tumor functions of macrophages, leading to the secretion of various tumor-inhibiting factors such as NO and reactive oxygen intermediates (Corthay et al., 2005). Besides, INF-γ inhibits tumor growth by inducing the production of anti-angiogenic chemokines derived from tumor cells or stromal cells that exist in the TME (Qin and Blankenstein, 2000). C-C chemokine receptor type 7 (CCR7) is up-regulated in dendritic cells exposed to LL-37 that causes infiltration of CD8^+^ T-cells into tumor sites (Roberts et al., 2016). Increased infiltration of activated CD8^+^ T-cells into tumors following LL-37 exposure could be considered a favorable clinical outcome in tumor regression (Findlay et al., 2019). LL-37 has also been shown to inhibit TGF-β1 and IGF-1–induced collagen synthesis in fibroblasts that could interfere with fibroblast-supported cancer cell proliferation (Zhang M. et al., 2019). Collectively, AMPs could affect the immune system, eliminate cancer cells, and prevent tumor growth by recruiting different immune system components.

### Reducing Multidrug Drug Resistance

Multidrug resistance has remained a considerable bottleneck in cancer treatment. Cancer cells have developed numerous resistance mechanisms to overcome the toxic effects of chemotherapeutic agents. One of the most studied mechanisms is the transmembrane ATP-binding cassette (ABC) transporter superfamily, which enhances the efflux of several chemotherapeutic drugs. In this regard, the pivotal role of P-glycoprotein (P-gp/ABCB1), as a member of the ABC superfamily, has been most well-known (Zhang H. et al., 2021).

AMPs reduce the MDR in some cancer types, such as acute myeloid leukemia (AML), glioblastoma, and urinary bladder cancer. This ability has encouraged clinician-scientists to use AMPs as a combination therapy with conventional chemotherapeutic drugs, such as temozolomide and cytosine arabinoside (Jafari et al., 2022). Some previous studies have shown the role of ROS in reducing MDR and the negative correlation between ROS levels and P-gp expressions (Pandey et al., 2011; Lo and Wang, 2013). Interestingly, AMPs could increase ROS in cancer cells and reduce MDR in some cancer types. For instance, hepcidin, which is secreted from MSCs, increases the anti-neoplastic effects of chemotherapeutic agent epirubicin by enhancing ROS generation and reducing ABC pumps in human squamous cell carcinoma and cervical cancer cells (Lo et al., 2015; Juang et al., 2016). This potential effect of AMPs against MDR encourages scientists to consider combining therapeutic approaches consisting of conventional chemotherapeutic regimens with AMPs. The summary of anti-neoplastic results of MSC-derived AMPs is shown in Table 2.

**TABLE 2:** Anti-neoplastic effects of MSC-derived AMPs.

Mechanism	AMP	Affected factors	Effects	References
Apoptosis and cell death	LL-37	Cell membrane	-Membrane disruption	Xhindoli et al. (2016)
	Defensins			Nguyen et al. (2011)
	Hepcidins			
	LL-37	AIF	-Mitochondrial ΔΨm dissipation	(Mader et al., 2009)
			-Increasing the translocation of AIF into the nucleus	
	LL-37	APAF1	Cleaving and activating caspase-9	(Li et al., 1997; Mader et al., 2009)
	LL-37	Bax	-Activation of the intrinsic pathway of apoptosis	(Mader et al., 2009; Sevrioukova, 2011)
	LL-37	Cathepsins	-Augmenting lysosomal membrane permeability	Mader et al. (2009)
	Hepcidin	ROS	-Induction of DNA damage	(Lo et al., 2015)
			-Increasing proapoptotic factor	
	Hepcidin	c-Jun	-Downregulation of c-Jun	Chen et al. (2009)
			-Increasing TP53	
	LL-37	Fructose 6-phosphate	-Suppresses ATP generation	Kuroda et al. (2015)
Inhibiting Proliferation	LL-37	miR-663a	Activating p21	(Kuroda et al., 2017)
	LL-37	BMP4	-Inducing p21 activation	(Wu et al., 2010)
			-G1/S proliferation phase transition delay	
	LL-37	TP53	-Affecting TME	(Orr et al., 2003; Cheng et al., 2015b; Sahai et al., 2020)
			-Inducing G2/M proliferation phases arrest	
Angiogenesis Inhibition	Defensins	VEGF Integrins	-Inhibit the migration of endothelial cells	Kougias et al. (2005)
			- Blocking av- or β1-integrin	
	LL-37	NR	-Reducing MVD	Fan et al. (2015)
	LL-37	Cell membrane	- Vascular smooth muscle cell apoptosis	Ciornei et al. (2006)
Immunomodulation	LL-37	ROS	-Increasing ROS production in neutrophils	(Esfandiyari et al., 2019; Wu et al., 2019)
	LL-37	IFN-γ	-Activating the anti-tumor functions of M1 macrophages	(Mookherjee et al., 2009; Fabisiak et al., 2016)
		IFN-β		
		IFN-α		
	LL-37	CCR7	-Increasing the infiltration of activated CD8^+^ T-cells into the tumor site	(Findlay et al., 2019)
	Lcn-2	NR	-M2 to M1 polarization	Cheng et al. (2015a)
Reducing MDR	Hepcidin	ROS	-Reducing efflux pumps	(Lo et al., 2015; Juang et al. (2016)

APAF1, Apoptotic protease activating factor 1; ROS, reactive oxygen species; miR-663a, microRNA-663a; BMP4, bone morphogenetic protein 4; TP53, tumor protein 53; IFN, Interferon; CCR7, C-C chemokine receptor type 7; MDR, Multidrug resistance; NR, not reported

## The Role of Preconditioning in Antimicrobial Peptides-Based Therapeutic Approaches

The cell’s microenvironment significantly affects its biological exclusivities such as viability, proliferation, metabolism, differentiation, migration, and paracrine activity (Sart et al., 2014; Shafiq et al., 2016). Following this fact, a strategy to improve cells’ therapeutic activities was developed named “preconditioning,” defined as culturing cells under specific conditions or with particular substances to augment their advantageous biological properties. Various methods are available to instate cell preconditioning, including hypoxic preconditioning, pharmacological agents and biologic mediators, physical preconditioning, and acidic preconditioning (Moeinabadi-Bidgoli et al., 2021). However, in the context of AMPs, pro-inflammatory mediators, some growth factors, and vitamin D play more prominent roles in preconditioning approaches.

The TME is a complex milieu due to chronic immune cells’ infiltration, ischemia, glucose and oxygen tension, hyperthermia, oxidative stress, and inflammation (Whiteside, 2008). Preconditioning with inducers resembling the TME properties could boost some anticancer agents’ production, including AMPs.

The expression of some AMPs, including MSCs-derived AMPs, is enhanced in response to inflammatory cytokines (Kim et al., 2003). It is shown that preconditioning of hepatoma cells with IL-6 or IL-22 enhances hepcidin production by these cells (Armitage et al., 2011). Culturing human keratinocytes with TNF-α induces the expression of hBD-2 and LL-37 (Kim et al., 2009). Preconditioning of lung epithelial cells with TNF-α results in an augmented hBD-2 concentration in the culture plate (Harder et al., 2000). In a study, it has been observed that preconditioning of human alveolar adenocarcinoma with IL-17 plus TNF-α resulted in induction of Lcn production (Karlsen et al., 2010). IL-1 is a pro-inflammatory cytokine increased in TME through a positive feedback loop between cancer cells and immune cells (Gelfo et al., 2020). IL-1α and IL-1β preconditioning of lung epithelial cells and keratinocytes induce hBD-2 and Lcn generation (Harder et al., 2000; Cowland et al., 2003) (Hiroshima et al., 2011).

A strategy to boost AMP production is exposure to live bacteria and bacterial components. As elucidated, in the presence of *E. coli*, MSCs generate more significant amounts of LL-37 and hBD-2 (Krasnodembskaya et al., 2010; Sung et al., 2016). A study showed that exposure of human tracheobronchial epithelial to LPS up-regulates the expression of hBD-2 through stimulating CD14 pattern recognition receptors (Becker et al., 2000). It is also demonstrated that LPS preconditioning of macrophages increases their Lcn production (Meheus et al., 1993).

Since the 19th century, the crucial role of vitamin D in combating infections has been identified. Recent studies demonstrated that vitamin D triggers the expression of AMPs, specially cathelicidin. Moreover, low vitamin D serum level has been linked to increased cancer risk, a fact that we believe at least partially is associated with vitamin D influence on AMP generation (Gombart, 2009). A study has observed that higher vitamin D serum levels of psoriatic patients correlated with higher cathelicidin and hBD-2 amounts (Kim et al., 2010). Most notably, some preconditioning agents require vitamin D receptors and signaling activation to boost AMP production. For instance, it has been revealed that IFN-γ preconditioning of human macrophages enhanced cathelicidin generation only in vitamin D-sufficient sera (Fabri et al., 2011).

In summary, AMPs are effective biologic products to protect cells against various risks such as infections and neoplasm formation. Cell preconditioning with different inducers could be an ideal method to boost AMP production of MSCs as potential anticancer agents.

## Potential Clinical Applications of Antimicrobial Peptides in Cancer and Their Challenges

The results from pre-clinical studies utilizing AMPs demonstrated that these peptides could be used to treat infectious diseases. Multiple AMPs such as pexiganan acetate (MSI-78), which is the first commercially developed AMP, hLF1–11, CZEN-002, and novexatin (NP-213) have been approved for clinical use in various infectious diseases as alternatives to antibiotics (Moore, 2003; Gordon et al., 2005; Velden et al., 2009; Fjell et al., 2011). These approvements encourage utilizing AMPs in medical oncology as an alternative or combined with chemotherapeutic agents. In order to investigate the anti-neoplastic effects of AMPs, some phase clinical trials are ongoing or completed at www.clinicaltrials.gov. In a newly constructed clinical trial, Amaria et al. tried to find the optimal biological dose (OBD) of LL37 for the treatment of melanoma. They also evaluated the effect of LL37 on the immune system, especially T-cell responses and IFN-α expression at the treated tumor site to support control of the neoplasm (NCT02225366). However, the clinical anti-neoplastic effects of some other MSCs-produced AMPs such as hepcidin and hBD-2 have not been evaluated yet and need further studies.

Despite the remarkable therapeutic potential of AMPs, these agents are still at an early stage of technological maturation. Therefore, several challenges and drawbacks limit the commercial success of the pharmacological design of AMPs that could fit the market (Li et al., 2017; Jiang et al., 2021). However, using MSCs as an AMP producing system alongside a targeted delivery platform can bypass several bottlenecks of using AMPs as anti-neoplastic agents. The predominant concerns, especially in systemic administration, lie ahead in systemic toxicity, susceptibility of the peptides to protease degradation, short half-lives *in vivo*, and rapid kidney clearance (Zaiou, 2007; Kumar et al., 2018; Divyashree et al., 2020). Besides, several serum components such as negatively charged albumins, iron, and high- and low-density lipoproteins (HDL, LDL) could also affect the activity of AMPs (Schweizer, 2009; Huan et al., 2020). For example, It has been reported that the anti-tumor and antibacterial activities of human defensins are diminished by serum LDL (Zhong et al., 2021). As another concern, these peptides may show enormous toxic side effects on mammalian cells in their long-term use, such as hemolytic activity, inhibition of cell growth, cytotoxicity of host cells, and immunogenicity that limit their clinical applications (Roudi et al., 2017; Lei et al., 2019). As the last challenge, the high cost of synthesizing and producing these peptides determines the clinical and commercial development of AMPs on a large scale.

Utilizing MSCs as a targeted AMP delivery system can resolve many challenges of administering AMPs in cancer patients. Considering the fact that MSCs produce and release these peptides, AMPs would bypass the destructive effects of serum proteases, immune system, and rapid renal clearance effects. Previous studies have used MSCs as chemotherapeutic drug carriers to increase treatment efficacy by boosting tumor targeting (Babajani et al., 2020). MSCs also could protect AMPs against neutralizing effects of serum proteins and lipoproteins. MSCs produce and release AMPs under specific conditions like inflammation in the TME (Silva-Carvalho et al., 2021). In the long-term administration, this controlled release system would prevent toxic side effects on normal host cells. Besides, assuming MSCs as a biological factory of AMPs that is able to home near the primary and secondary tumors sites to release AMPs in a controlled manner could considerably reduce the high cost of synthesizing and producing these peptides. As another advantage, the antibacterial, antiviral, and antiparasitic effects of AMPs make them an appropriate choice for use in cancer patients. Becaause cancer patients are prone to high risk of infection due to immune system suppression related to administering various chemotherapeutic agents, bone marrow suppression, and the natural behavior of neoplastic cells, using AMPs may prevent or treat infectious diseases besides the anti-neoplastic effects (Grabowski et al., 2021).

## The Role of Exosomes in Delivery of Antimicrobial Peptides to Cancer Cells

Mesenchymal stem cells release their AMPs mainly in two distinctive methods: free (soluble) AMPs and exosome-packaged AMPs (Krasnodembskaya et al., 2010; Raghav et al., 2021).

Numerous studies have shown that MSCs secrete AMPs in response to infections and lesions. MSC release AMPs such as LL-37, hepcidin, and defensins in a soluble form as a part of innate immune system components to battle cancer cells and bacteria. Although the soluble form of agents could provide a notable concentration at the release site, they often lack targeting ability and are negligibly bio-persistent (Harman et al., 2017; Das et al., 2019; Esfandiyari et al., 2019). Considering targeting features of exosomes, AMPs delivery through an exosome-packaged system seems a desirable method to increase the therapeutic efficacy of these peptides.

**FIGURE 1:**
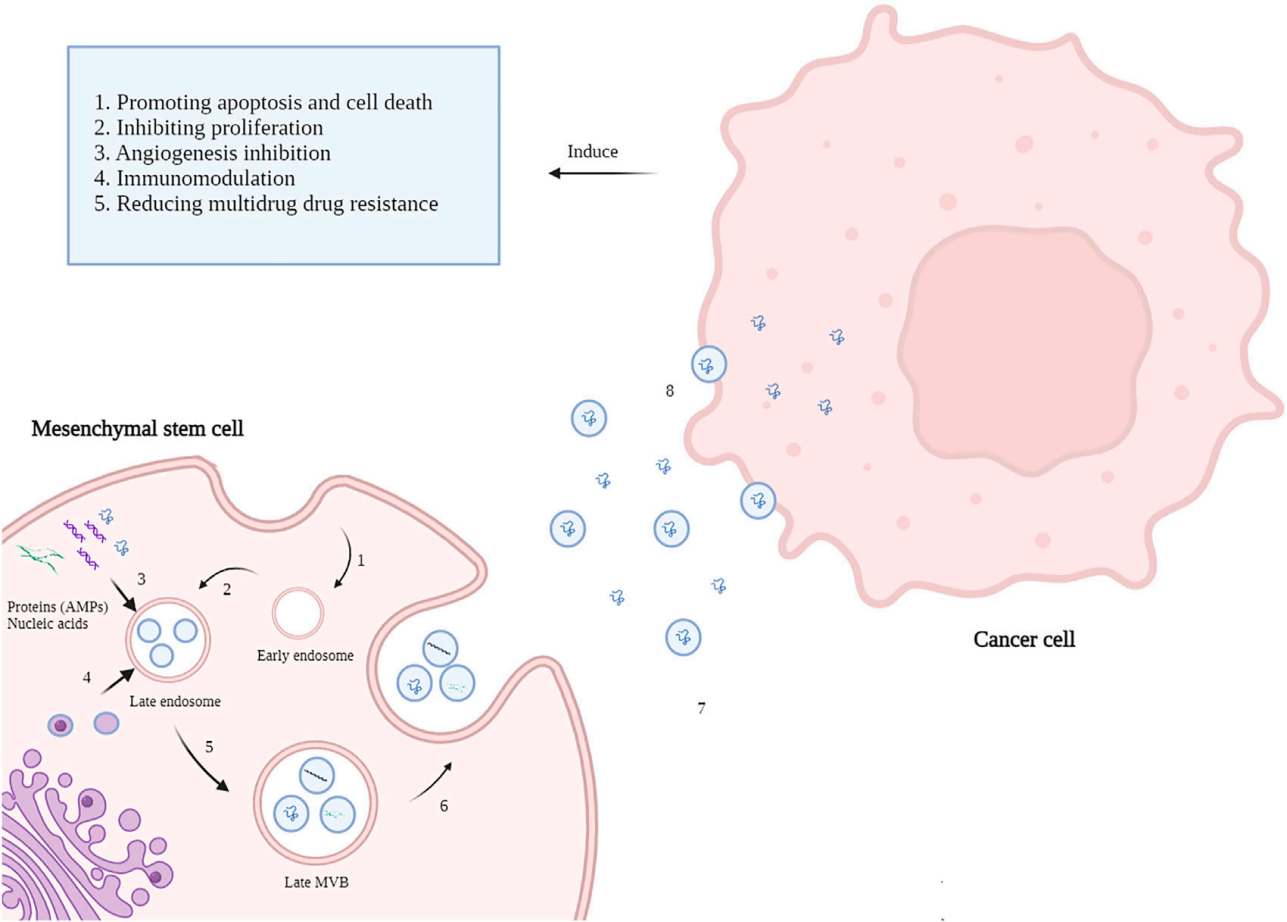
Mechanisms of MSC-derived AMPs delivery to cancer cells. 1. The Inward budding of the MSCs membrane creates an early endosome. 2. Early endosomes then progress to late endosomes when intraluminal vesicles (ILVs) incorporate lipids, nucleic acids, and proteins like AMP appear. 3. Cellular contents of MSCs such as AMPs, MicroRNAs, and lipids enters late endosomes via inward budding of the endosomal membrane. 4. Late endosome cooperates with Golgi apparatus mutually. 5. Incorporation of cellular content finally forms multivesicular bodies (MVBs). 6. MVBs fuse with the MSCs plasma membrane and release the vesicular contents called exosomes. 7. Exosomes carry AMPs toward cancer cells alongside the soluble AMPs in the tumor microenvironment. 8. Exosomes transfer their content to the cancer cells and induce anti-neoplastic effects (created by biorender.com).

Alongside the anti-neoplastic effects of MSCs, the MSCs-derived exosomes have also been widely studied regarding their considerable anticancer effects. Exosomes are a class of extracellular vesicles (EVs) with an average size of 30–150 nm released by nearly all cell types (Nawaz, 2017; Keshavarz Alikhani et al., 2021). Exosomes are generated *via* a process of inward budding in early endosomes and then are secreted *via* exocytosis into the extracellular microenvironment to facilitate cell-to-cell communication (Figure1).

First, it had been thought that exosomes are only a repository of cell waste, but then it was elucidated that they participate in various biological actions such as intercellular communication via the transfer of lipids, proteins, DNA, RNAs, and microRNAs (Gurunathan et al., 2021; Yousefi Dehbidi et al., 2021). Most MSC-induced biological effects are attributed to their paracrine activity, and it has been elucidated that exosome are the main component of cells’ paracrine elements. In this regard, exosome destruction via ultrasonication significantly diminishes cell-based therapeutic impacts (Namazi et al., 2018; Álvarez-Viejo, 2020). Various studies have reported that exosomes could be a targeted-delivery tool as they can incorporate bioactive molecules, promotes their stability, and carry them into specific tissues (Kim et al., 2016; Hu et al., 2020). Some studies have shown the anti-neoplastic influences of exosomes. For instance, MSC-harvested exosomes could limit ovarian cancer cells’ growth and colony formation by up-regulating mitochondria-mediated apoptosis factors, including BAX, caspase-3, and caspase-9, and consequent induction of cell cycle arrest and apoptosis (Reza et al., 2016). It has been demonstrated that MSC-originated exosomes significantly induce hepatocellular carcinoma suppression in rat models through enhancing chemosensitivity (Lou et al., 2015).

Some studies have shown that exosomes from different sources contain AMPs produced by the parent cell. It has been demonstrated that human sweat collected after an aerobic exercise contains exosomes enriched with AMPs such as cathelicidin, cathepsin B, lactoferrin, dermcidin, and defensin. These AMPs are encapsulated in sweat exosomes and participate in skin immune homeostasis (Wu and Liu, 2018). Urine, another body fluid, possesses exosomes that function as innate immunity components. These exosomes contain AMPs, including calprotectin and dermcidin (Hiemstra et al., 2014). Honey has been a traditional antimicrobial agent used to treat infected wound since ancient times (Giusto et al., 2017). It has been elucidated that gastrointestinal epithelium-derived exosomes carry AMPs, including hBD-2 and cathelicidin-27 to participate in luminal defense systems against microbial pathogens (Hu et al., 2013).

Mesenchymal stem cells communicate with the nearby and far cells through secreting exosomes that contain MSC-derived AMPs (Marrazzo et al., 2021). It is elucidated that direct injection of stem cells into the host may be associated with numerous risks such as teratoma and tumor formation, massive grafted cell death, vascular obstruction, and immune reaction. In contrast, exosome administration is almost free of these hazards. Moreover, exosomes could exert approximately the same therapeutic impacts as their parent cells. It is believed that the therapeutic effects of MSCs are mainly due to their exosome secretion (Huang et al., 2017; Zhang H. et al., 2019; Takeuchi et al., 2019). Furthermore, exosomes possess significantly advantageous properties such as low immunogenicity, biocompatibility, biodegradability, long-term storage capacity, and prolonged circulation time (Ma et al., 2017; Yousefi Dehbidi et al., 2021). These properties bring up exosomes as an excellent tool for AMP delivery.

Antimicrobial peptide-containing exosomes could be modified to induce more significant anti-neoplastic influences. Various chemotherapeutic drugs such as gemcitabine, doxorubicin, and paclitaxel were loaded successfully into exosomes and demonstrated potent anticancer activity. So, as a future distinction, chemotherapy agents could be loaded in MSC-derived exosomes alongside AMP content to promote their anti-neoplastic impacts (Tian et al., 2014; Salarpour et al., 2019; Li et al., 2020). Exosomes could also be engineered to target tumor sites, resulting in improved antitumor activity. Considering the fact that tumor cells express specific surface molecules, exosomes will have increased delivery potency into the tumor site via conjugating tumor-targeting ligands on the exosome’s surface. In addition, TME possesses particular characteristics such as low pH (Jamshidifar et al., 2021). By incorporating particular molecules into the exosome’s structure, they will be able to release their cargo (including AMPs) at a higher rate in the acidic microenvironment of the TME and induce more excellent anti-neoplastic effects (Lee and Kim, 2019; Lin et al., 2019; Nie et al., 2020).

**FIGURE 2:**
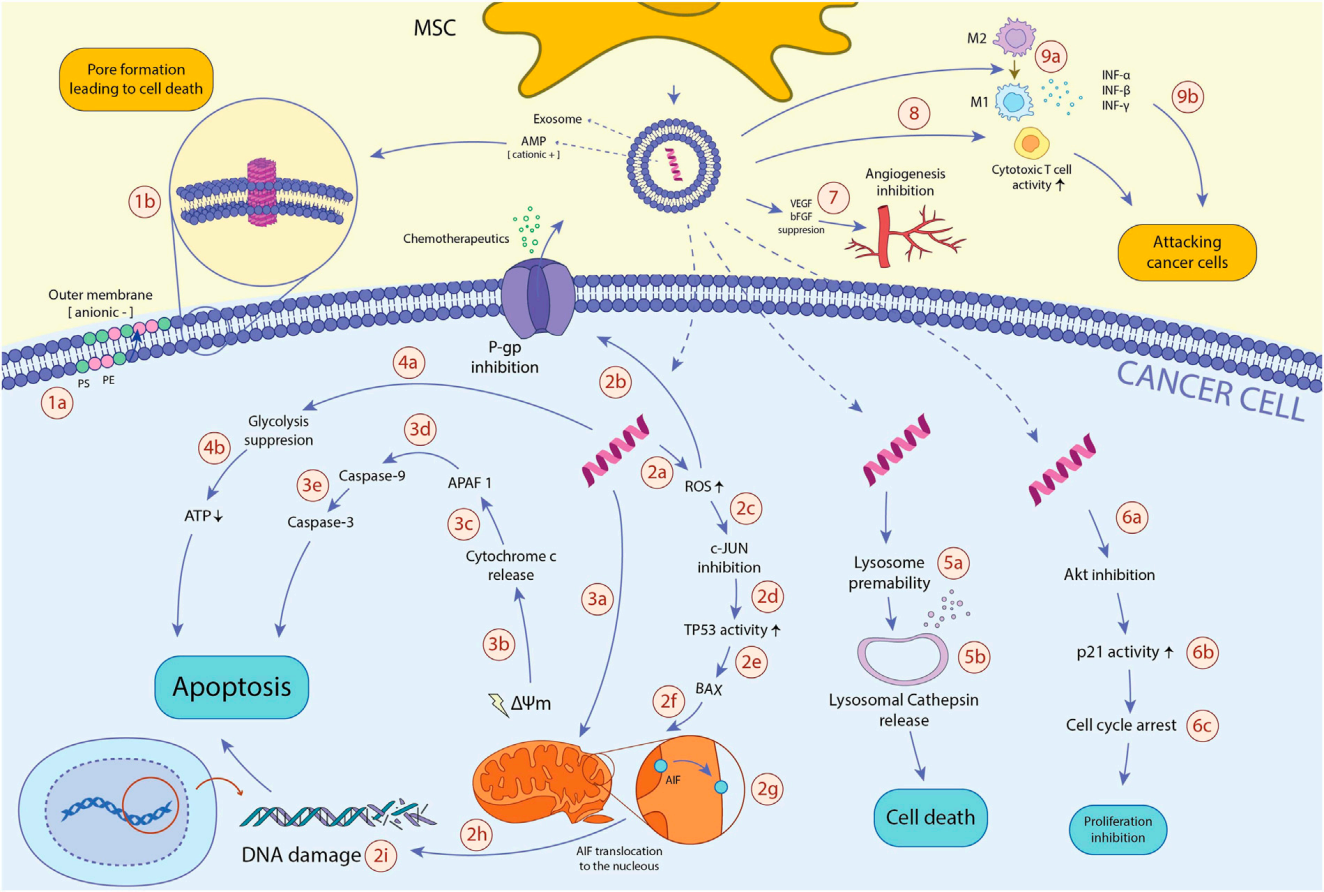
The anti-neoplastic effects of MSCs-derived AMPs. AMPs reduce the viability of cancerous cells through various mechanisms: 1a. In TME, hypoxia and excessive ROS amounts induce translocation of PS and PE from the inner membrane to the outer membrane of the cancer cell, resulting in the anionic charge of the outer membrane and subsequent incline of the cationic AMPs. 1b. Cancer cell membrane-AMP interaction leads to membrane dysregulation, pore formation, and ultimately, cancer cell death. 2a. After entering AMP to the cancer cell, it promotes intracellular ROS production. 2b. Excessive ROS amount inhibits P-gp activity, a pump playing an essential role in chemotherapeutics efflux and instating MDR, resulting in the enhanced vulnerability of cancer cells to chemotherapeutic drugs. 2c. High ROS levels also hamper c-JUN activity. 2days. When c-JUN inhibitory impacts on the TP53 tumor suppressor gene abrogates, TP53 function will be enhanced. 2e. TP53 profoundly induces BAX expression. 2f. BAX translocates to mitochondria. 2g. in the mitochondria, BAX triggers mitochondrial membrane potential (ΔΨm) dissipation and AIF translocation from the inner membrane to the outer membrane. 2h. AIF transfers to the nucleus. 2i. In the nucleus, AIF binds to DNA, causes DNA damage, and ultimately programmed cell death of the cancer cell. 3a. AMP disrupts mitochondrial membrane, leading to mitochondrial membrane degradation, mitochondrial swelling, and damage. 3b. Consequently, AMP dysregulates the mitochondrial membrane potential (ΔΨm), which leads to cytochrome c release. 3c. Cytochrome c activates APAF1. 3days. APAF1 activates caspase-9 pro-enzyme and induces its translocation into the cytoplasm. 3e. Activated caspase-9 ultimately triggers caspase-3 activity, one of the main enzymes through the apoptosis process. 4a. AMPs alter the cancer metabolic activity and inhibit glycolysis, the primary process responsible for ATP generation in cancer cells (known as The Warburg effect). 4b. glycolysis inhibition results in ATP depletion, which leads to cancer cell death. 5a. AMPs also augment lysosomal membrane permeability. 5b. Increased lysosomal permeability leads to the release of lysosomal cathepsin into the cytosol, which finally initiates cytosol death signaling pathways. 6a. AMP downregulates Akt expression. 6b. downregulating Akt expression leads to enhanced p21 activity. 6c. p21 induces cell cycle arrest, leading to the diminished proliferation of the cancer cell. 7. AMPs hamper tumor-associated angiogenesis via inhibiting the function of bFGF and VEGF pro-angiogenic factors. 8a. AMPs promote the activity of cytotoxic T cells, which ultimately leads to enhanced immune system activity against cancer cells. 9a. AMPs boost macrophages’ shift to anti-cancer M1 phenotype. 9b. M1 macrophages suppress tumor growth through phagocytosis and cytokine secretion such as IFN-γ, IFN-β, and IFN-α. Abbreviations: AMP, antimicrobial peptide; TME, tumor microenvironment; ROS, reactive oxygen species; PS, phosphatidylserine; PE, phosphatidylethanolamine; P-gp, P-glycoprotein 1; TP53, tumor protein 53; BAX, Bcl-2 Associated X protein; AIF, apoptosis-inducing factor; APAF1, apoptotic protease activating factor 1; Akt, phosphorylated protein kinase B; bFGF, basic fibroblast growth factor; VEGF, vascular endothelial growth factor; IFN: interferon.

Because MSC-derived exosomes contain AMPs and possess anti-neoplastic activity through various mechanisms, AMP content may play a critical role in anticancer features of exosomes. Furthermore, exosomes could be an ideal delivery tool for AMPs as they improve AMPs’ biocompatibility, circulation time, and other biological yardsticks necessary for successful delivery and efficacy. However, further studies are needed to elucidate the role of AMP-content of MSC-derived exosomes in their anticancer effects. The anti-neoplastic mechanisms of MSCs-derived AMPs, as well as exosome delivery of these peptides to the cancer cells, are shown in Figure2.

## Conclusion

Mesenchymal stem cells induce considerable anti-neoplastic effects by enhancing cancer cell apoptosis and cell cycle arrest. They also reduce angiogenesis, multidrug resistance, and inflammation in the tumor microenvironment. To the best of our knowledge, MSCs also release some AMPs such as LL-37, hepcidin, hBD-2, and Lcn2, which have anti-neoplastic features. Since MSCs produce and release AMPs, it could be considered that AMPs are a part of the anti-neoplastic effects of MSCs. However, further studies are needed to shed light on the anticancer effects of MSCs-derived AMPs.
